# Analysis of ^226^Ra content and ^222^Rn exhalation rates in soil samples from Wukro, Tigray, using SSNTDs

**DOI:** 10.12688/f1000research.160449.1

**Published:** 2025-03-13

**Authors:** Nigus Alene Assefa

**Affiliations:** 1 Department of Physics, CNCS, Mekelle University, Mek'ele, Tigray, Ethiopia

**Keywords:** Rn exhalation rates, effective Ra content, sealed can technique, Alpha index

## Abstract

**Background:**

Radon gas, a decay product of radium, is a significant environmental health risk due to its radioactive properties. Understanding the radium content and radon exhalation rates in soil is crucial for evaluating potential radiological hazards and ensuring environmental safety.

**Methods:**

Soil samples were collected from twelve different locations in Wukro, Tigray, representing various soil types. The sealed can technique, using LR-115 Type-II plastic track detectors, was employed to measure radium concentrations and radon exhalation rates over a four-month exposure period. Radon mass exhalation rates, surface exhalation rates, and radium concentrations were calculated, and the Alpha Index was also determined to assess radiological risk.

**Results and Discussion:**

The radon mass exhalation rates ranged from 0.18 × 10 ^−7^ Bq.kg ^−1^.d ^−1^ to 0.82 × 10 ^−7^ Bq.kg ^−1^.d ^−1^, with a mean of 0.48 × 10 ^−7^ Bq.kg ^−1^.d ^−1^. Surface exhalation rates varied from 0.38 × 10 ^−6^Bq.m ^−2^.d ^−1^ to 1.72 × 10 ^−6^ Bq.m ^−2^.d ^−1^, averaging 1.02 × 10 ^−6^ Bq.m ^−2^.d ^−1^. Radium concentrations ranged from 0.33 to 1.47 Bq.kg ^−1^, with an average of 0.87 Bq.kg ^−1^. A significant positive correlation between radium content and radon exhalation rates was observed, indicating a direct relationship between these variables. Clay soils exhibited the highest radium concentrations, while sandy soils had the lowest. All measured values were below the safety limit of 370 Bq.kg ^−1^ recommended by OECD and UNSCEAR, suggesting no significant radiological risk in the study area.

**Conclusion:**

This study highlights the importance of monitoring natural radiation levels for environmental safety. The findings provide a baseline for future studies and emphasize the need for continuous assessment to detect any long-term changes in soil radioactivity.

## 1. Introduction

Radium ( ^226^Ra), a naturally occurring radioactive element that exists as a solid under standard temperature and pressure, is generated as a result of uranium's decay process. As radium undergoes decay within the soil, it produces radon isotopes, which initially diffuse into air-filled pores of the soil matrix. The speed at which radon escapes from the soil into the atmosphere is referred to as the radon exhalation rates. As demonstrated in previous studies by Al-Saadi et al. (2015), Kumar and Narang (2014) and Elzain (2015), the exhalation rates can be measured either per unit area or per unit mass of the soil samples. Radon, a colorless and odorless radioactive gas, is constantly being generated by radium present in rock, soil, water and materials derived from rocks. This radioactive gas is found everywhere and cannot be avoided. The extensive prevalence of its parent elements and its prolonged half-life contribute to significant adverse impacts on human health. Radon has been officially recognized as an occupational respiratory carcinogen by an international research agency, being classified as a carcinogenic substance. IARC has classified radon as a carcinogenic agent, officially recognizing it as a major occupational respiratory hazard and regarded as the second leading cause of lung cancer globally, following tobacco smoking ( Kakati et al., 2013 and S. Monica and Jojo, 2017). Hence, the assessment of radon levels in the environment, particularly in soil, is essential from a public health standpoint. Accurate measurements of radium contents and exhalation rates in soil samples can provide valuable insights into the potential radiological hazards in a given area. In this study, investigations have been conducted to evaluate the ^226^Ra content and ^222^Rn exhalation rates in soil samples collected from Wukro town in Tigray regional state, utilizing type II LR-115 plastic detector. The primary objectives of this study were to assess the concentration of radium and determine the mass and surface-based radon exhalation rates. And, it will contribute to a better understanding of radon distribution in the region and its implications for environmental safety and public health.

## 2. Site description of Wukro town

The town is situated at an elevation of approximately 1,972 meters above sea level, offering a unique highland climate. Its geographical coordinates (latitudes and longitudes) are 13°47′N and 39°36′E respectively, placing it in a region characterized by diverse geological formations and varied soil types.

## 3. Methods

### 3.1 Sample collection

Soil samples were collected from twelve different locations in Wukro town, Tigray, using the grab sampling method to ensure variability across the study area. The study locations were chosen to represent the diversity in geological formations and soil types in the region. The samples were obtained from the upper 30 cm of the soil, which is typically where radium content and radon exhalation rates are most significant. The locations covered different soil types, including silt, clay, and sand, as outlined in Table 1.

**Table 1.** ^226^Ra contents and radon exhalation rates of the study area.

Detector code	Soil types	Corrected track density ρ (tracks.cm ^−2^)	Effective radium content (Bq.kg ^−1^)	Exhalation rates		
				Mass exhalation (Bq.kg ^−1^d ^−1^) Ex (M) ×10 ^−7^	Surface exhalation (Bq.m ^−2^d ^−1^) Ex (S) ×10 ^−6^	Alpha index I _α_
SI-1	Silt	90.00	0.99	0.55	1.15	0.009
CL-2	Clay	133.30	1.47	0.82	1.72	0.014
CL-3	Clay	120.00	1.32	0.74	1.55	0.013
SA- 4	Sand	30.00	0.33	0.18	0.38	0.003
SI- 5	Silt	80.00	0.88	0.49	1.03	0.008
SA- 6	Sand	36.70	0.40	0.22	0.46	0.004
SA- 7	Sand	43.30	0.48	0.27	0.57	0.004
SA- 8	Sand	50.00	0.55	0.31	0.65	0.005
CL- 9	Clay	116.70	1.28	0.72	1.51	0.013
CL-10	Clay	110.00	1.21	0.68	1.43	0.012
SI- 11	Silt	73.30	0.81	0.45	0.95	0.008
SI- 12	Silt	66.70	0.73	0.41	0.86	0.007
Min.	-	30.00	0.33	0.18	0.38	0.003
Max.	-	133.30	1.47	0.82	1.72	0.014
Mean	-	79.20	0.87	0.48	1.02	0.008
SD	-	33.70	0.37	0.21	0.44	0.004

**Figure 1.**
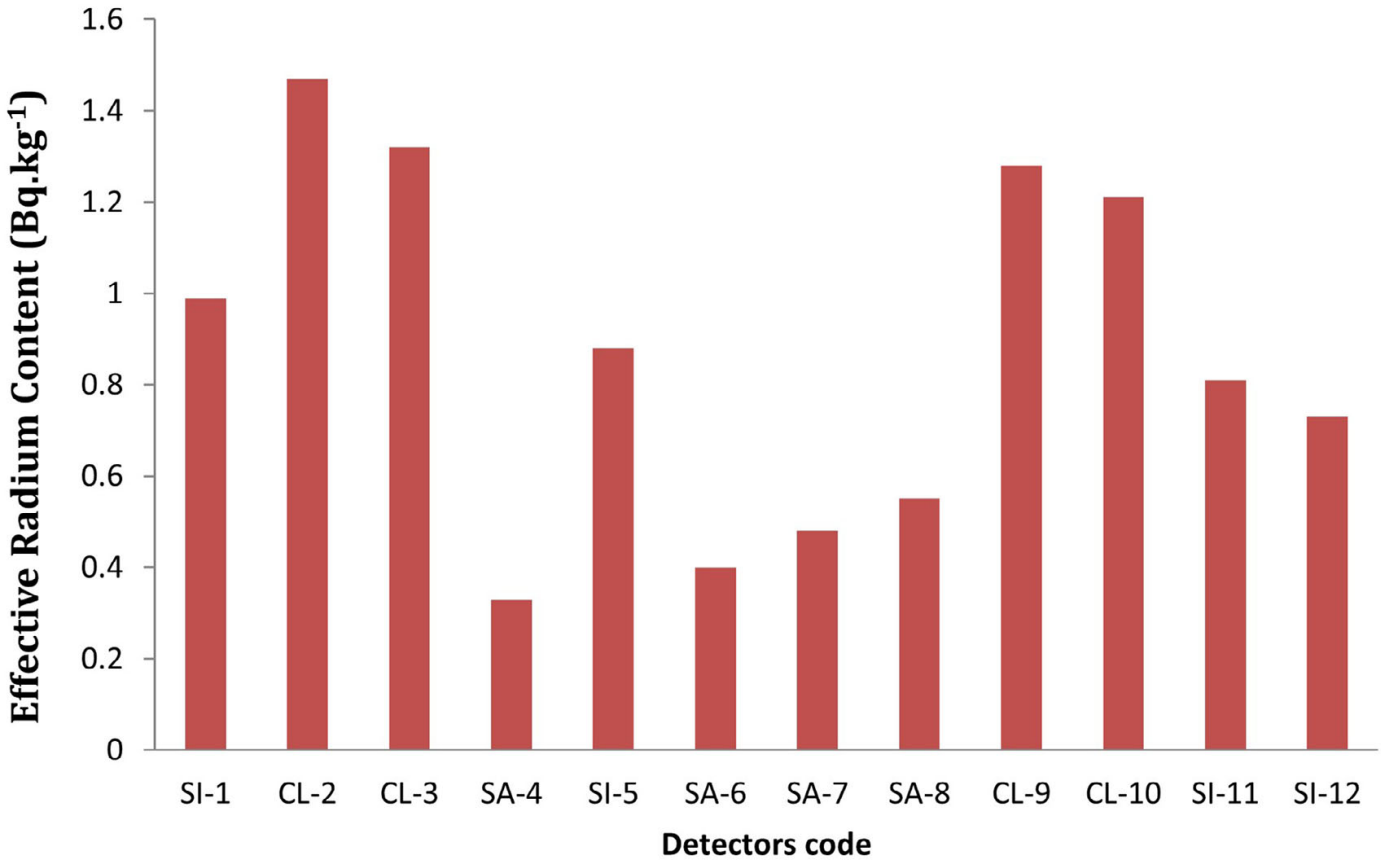
^226^Racontent variations at different sites.

**Figure 2.**
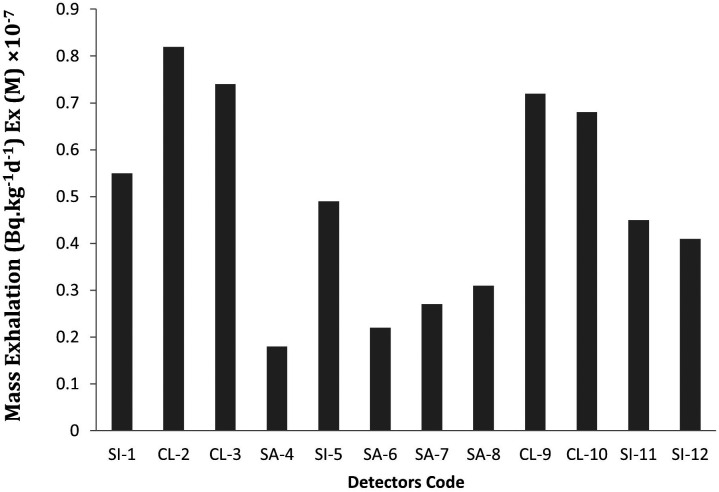
^222^Rn exhalation rate in terms of mass at different locations of the study area.

**Figure 3.**
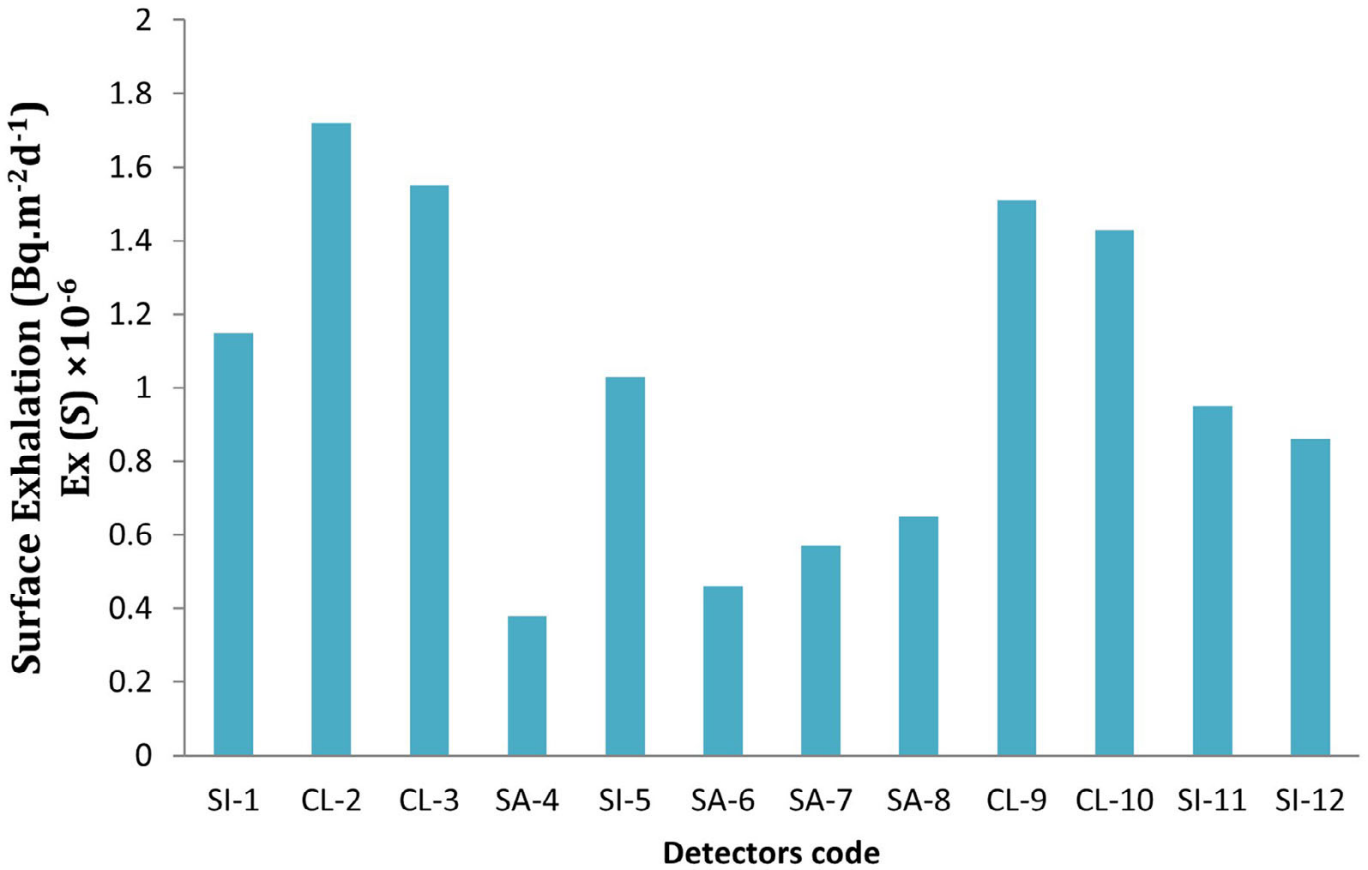
^222^Rn variation in terms of surface.

### 3.2 Sample preparation

Upon collection, soil samples were dried at room temperature and crushed into fine powder to ensure uniformity and consistency across all samples. The powdered soil was then sieved using a 200-micronmesh to eliminate larger particles and ensure that only fine particles were used for the subsequent measurements. For each measurement, 200 grams of the fine soil powder was placed into a cylindrical can (dimensions: 8 cm height × 12 cm diameter). The can was then sealed tightly to prevent any loss of radon gas and stored in a controlled environment for a period of four months to allow the radon to reach equilibrium with its decay products.

### 3.3 Detector setup and exposure

A piece of LR-115 Type-II plastic track detector (dimensions: 2 cm × 3 cm) was affixed to the inner surface of each cylindrical can, positioned approximately 0.65 meters above the surface of the soil sample. The detectors were exposed to the radon gas released from the soil for a period of four months. This exposure allowed the radon to interact with the detector, creating tracks that could later be analyzed to determine the radon concentration.

### 3.4 Chemical treatment and track counting

After the exposure period, the LR-115 detectors were removed from the cans and chemically etched to reveal the tracks. The detectors were immersed in a solution of 2.5N NaOH at a temperature of 70°C for one and a half hours. This chemical treatment caused the detector material to dissolve along the track paths created by the alpha particles emitted by radon. The etched detectors were then examined under an optical microscope at a magnification of 400× to count the number of tracks, which correspond to the interactions between alpha particles and the detector surface.

### 3.5 Radium content and radon exhalation rate calculations

After closing the can, the concentration of radon within it begins to increase over time, following the relationship:

$$C_{\mathrm{Rn}}=C_{\mathrm{Ra}}\;\left(1-e^{-\mathrm{\lambda t}}\right)$$

Where, C _Ra_ represents the effective radium content of the sample.

The observed track density on the detector can be expressed as: ρ = KC _Ra_T _e_

Where K (K = 0.0245 tracks cm ^−2^d ^−1^ per Bqm ^−3^) is the sensitivity factor of the detector and Te is the effective exposure time accounts for the decay and can be calculated using the equation expressed by:

$$\mathrm{Te}=\left[T-1/\lambda_{\mathrm{Rn}}\left(1-e^{-\text{λRnT}}\right)\right]$$

C _Ra_ (effective radium content) of the soil sample can also be determined using the relation:

$$C_{\mathrm{Ra}}=\mathrm{\rho Ah}/{\mathrm{KT}}_{e}M$$

Where, ρ is the corrected track density on the detector

M represents the weight of sample in kilograms.

A is the cross sectional area of the cylindrical can in square meters, and h denotes the vertical distance between the detector and the surface of the sample, measured in meters.

Mass exhalation rate and the surface exhalation rate of radon from the soil samples are calculated using the expressions:

$$E_{x}\left(M\right)=C_{\mathrm{Ra}}\;\left(\lambda_{\mathrm{Ra}}/\lambda_{\mathrm{Rn}}\right)/T_{e}{\text{AndE}}_{x}\left(S\right)=E_{x}\left(M\right)\left(M/A\right)$$

In these equations:

*λ _Ra_* Represents the decay constant for Radium ( ^226^Ra) and *λ _Rn_* for decay constant of Radon ( ^222^Rn)

**Alpha index (I**_**α**_ **)**

The alpha index (I _α_) is a dimensionless parameter defined as I _α_=C _Ra_/200 is used to assess the radiological risk associated with radon exhalation from building materials, as discussed by ( Abdalsattar Kareem, 2013). The prescribed threshold for exemption of 226Ra concentration in building materials is 100 Bq/kg, whereas the suggested maximum allowable limit is 200 Bq/kg. If the Radium concentration in construction materials exceed 200 Bq/kg, the resulting exhalation rates could lead to indoor radon levels rising above 200 Bq/m ^3^ potentially creating healthy risks. While, if the radium activity concentration is below 100 Bq/kg, the corresponding radon concentration remains under 200 Bq/m ^3^, indicating minimal risk. These considerations are evident in the alpha index. The recommended threshold of ^226^Ra is 200 Bq/kg, for which I _α_ is set at 1 ( Rafique M et al., 2011).

## 4. Results and Discussion

Effective radium content in the soil samples varies from 0.33 to 1.47 Bq kg ^−1^, with a mean value of 0.87 Bq kg ^−1^. Maximum radium concentration was found in clay soils (e.g., CL-2: 1.47 Bq kg ^−1^), while the lowest was observed in sandy soils (e.g., SA-4: 0.33 Bq kg ^−1^). This value aligns with the general understanding that clay-rich soil types tend to retain more radium content due to their smaller particle sizes and lower permeability, which limit the mobility of radon. While, the sandy type of soils, with their higher permeability and larger particle sizes, allow for easier diffusion of radon, leading to lower radium concentrations ( M. S. A. Khan, 2015 and Nooreldin Fadol et al., 2016). Mass exhalation rates varied from 0.18 × 10 ^−7^ Bq. kg ^−1^.d ^−1^ to 0.82 × 10 ^−7^Bq. kg ^−1^.d ^−1^ with average value of 0.48 × 10 ^−7^Bq. kg ^−1^.d ^−1^ and the surface exhalation rates varies from 0.38 x10 ^−6^Bq. kg ^−1^.d ^−1^ to 1.72 × 10 ^−6^ Bq.m ^−2^d ^−1^ with a mean value of 1.02 × 10 ^−6^ Bq.m ^−2^d ^−1^. Maximum exhalation rates are observed in clay soils, which are likely due to the combination of higher radium concentrations and lower soil porosity, leading to greater radon accumulation in the soil and subsequent release when the radon gas diffuses to the surface. While, sandy soils, despite having lower radium content, show lower exhalation rates, which can be attributed to their higher porosity and more efficient radon escape pathways. A positive correlations supports the principle that radon exhalation is directly influenced by the availability of radium, which decays to produce radon gas ( OECD, 1979; UNSCEAR, 1993).

## 5. Conclusion

From the result it reveals that a significant variation in radium concentrations and radon exhalation rates based on the soil type, with clay soils exhibiting higher radium content and radon exhalation rates compared to sandy soils. It is a notable correlation between Ra content and Rn exhalation rates, and revealing that soils with higher radium concentrations tend to release more radon. The Alpha Index values indicating that the radon risk from these soils is minimal, if used in construction. Therefore, it can be inferred that, from a radium-related health perspective, the study area is free from hazards.

## Ethical statement

Ethical approval and consent were not required. This study did not involve human or animal subjects. Soil samples were collected in accordance with local regulations, and necessary permissions were obtained from the relevant authorities prior to sample collection. The research complies with ethical standards for environmental studies.

## Data Availability

Dryad: Analysis of 226Ra content and 222Rn exhalation rates in soil samples from Wukro, Tigray, using SSNTDs. Doi: https://doi.org/10.5061/dryad.n8pk0p35z ( Alene, 2025). The project contains the following underlying data:
• README.md •
Wukro_Soil_Radium_Radon_Data.xlsx Data are available under the terms of the Creative Commons Zero v1.0 Universal (CC0-1.0 universal)
