# Supplementary material for: Analysis of   226Ra content and 222Rn exhalation rates in soil samples from Wukro, Tigray, using SSNTDs
Source: F1000Res. 2025 Mar 13;14:289. [Version 1] doi: 10.12688/f1000research.160449.1 (PMC12371319; doi:10.12688/f1000research.160449.1)
Supplement: Peer review history

# Reviewer report: Reviewer response for version 1

**DOI:** 10.5256/f1000research.176353.r394575

**Published:** 2025-08-21

**Authors:** Tayseer I. Al-Naggar

**Affiliations:** 1 Najran University, Najran, Najran, Saudi Arabia

The scientific value of the study is diminished, and the capacity to contextualize environmental conditions for each site is limited when sample collection sites are not geographically representative.

 - A comprehensive map with GPS coordinates and obvious marks for samples with higher concentrations is advised. This will make spatial analysis easier in the future.

 Methodology and Discussion Section: The methodology section is too brief, which reduces reproducibility and transparency.

 - Details about analysis methods, detector types (e.g., CR-39 or alpha detectors), and laboratory conditions are missing.

 Although statistical connections between radon and radium concentrations are essential for comprehending the radiological source and geological context, they are not shown in the discussion.

 Improve the methodology section by adding a process flow diagram that shows the phases involved in analysis and measurement.

 For every radionuclide, provide exact results, including range values, averages, and standard deviations.

 Use graphics and statistics that examine the connection between radium and radon concentrations to bolster the conversation.

 To provide a more thorough scientific explanation, relate results to the geological characteristics of the sampled areas.

Is the work clearly and accurately presented and does it cite the current literature?

No

If applicable, is the statistical analysis and its interpretation appropriate?

No

Are all the source data underlying the results available to ensure full reproducibility?

Partly

Is the study design appropriate and is the work technically sound?

No

Are the conclusions drawn adequately supported by the results?

Partly

Are sufficient details of methods and analysis provided to allow replication by others?

No

Reviewer Expertise:

Nuclear and radiation physics

I confirm that I have read this submission and believe that I have an appropriate level of expertise to confirm that it is of an acceptable scientific standard, however I have significant reservations, as outlined above.

# Reviewer report: Reviewer response for version 1

**DOI:** 10.5256/f1000research.176353.r394569

**Published:** 2025-08-13

**Authors:** Najeba Farhad Salih

**Affiliations:** 1 Koya University, Erbil, Iraq

Dear Editor

 This manuscript  is good but need a lot of correction in the every thing, and the text need a lot of references

 because the number of references are very little, should be more than 25 references, try to check all comments and add new references, and your manuscript need a lot of correction.

 THANKS

*https://f1000research-files.f1000.com/linked/737838.160449_attachment.pdf*

Is the work clearly and accurately presented and does it cite the current literature?

Partly

If applicable, is the statistical analysis and its interpretation appropriate?

Yes

Are all the source data underlying the results available to ensure full reproducibility?

No

Is the study design appropriate and is the work technically sound?

No

Are the conclusions drawn adequately supported by the results?

Partly

Are sufficient details of methods and analysis provided to allow replication by others?

Partly

Reviewer Expertise:

My area study is are nuclear physics, medical physics, radiation protection and dosemetry.

I confirm that I have read this submission and believe that I have an appropriate level of expertise to confirm that it is of an acceptable scientific standard, however I have significant reservations, as outlined above.

# Reviewer report: Reviewer response for version 1

**DOI:** 10.5256/f1000research.176353.r373734

**Published:** 2025-04-15

**Authors:** Balvinder Singh

**Affiliations:** 1 Department of Physics, Gurugram University, Gurugram, Haryana, India

**General Comments**

 This manuscript presents a relevant and region-specific study on the radiological characteristics of soil, focusing on radium-226 content and radon-222 exhalation rates using solid-state nuclear track detectors (LR-115 Type II). The work addresses public health implications of naturally occurring radioactive materials (NORMs) and contributes valuable baseline data for Wukro, Tigray—a region with limited prior radiometric investigation.

 The paper is well-structured, methodologically sound, and aligned with current environmental safety research. However, several improvements are recommended to enhance the scientific clarity, reproducibility, and completeness of the study.

**Major Comments**

1. While the paper states that twelve locations were selected, the exact geographic coordinates of the sampling sites are not provided. Including a table or map with GPS coordinates would greatly enhance transparency and reproducibility. A simple site map showing sample distribution over Wukro town (with soil types annotated) would be beneficial.
2. Area covered in the study can be specified for the justification of the number of the samples.
3. The use of twelve samples is reasonable for a preliminary study. However, a brief justification of sample size—possibly referencing previous studies or variability of soil types—would help affirm its statistical and spatial representativeness.
4. Clarify why a 30 cm depth was selected. Justify the 4-month exposure period. Mention environmental conditions during exposure.
5. Include correlation coefficients between Ra and Rn. Discuss variability more explicitly.
6. Improve figure labeling and table formatting. Highlight min/max values for quick visual reference.
7. Use scientific notation consistently, e.g., 1.02 × 10⁻⁶ Bq·m⁻²·d⁻¹.
  Improve awkward phrasing like “And, it will contribute…”.
8. While the discussion rightly highlights higher radon exhalation in clayey soils due to lower porosity and higher radium retention, it would be beneficial to explore this variation in the context of the local geological formations. For instance, if certain sampling sites are underlain by radium-rich rocks such as granite or phosphate-bearing formations, this could significantly affect the radium content and subsequent radon release. Providing even brief geological descriptions of the sampled areas could strengthen the interpretation of spatial differences in exhalation rates.
9. The study would be further strengthened by a comparative analysis with similar studies conducted globally. Including a summary table or discussion that compares the measured radium concentrations and radon exhalation rates with reported values from other countries or regions (especially in similar geological settings) would contextualize the significance of the findings and highlight any anomalies or consistencies.

**Recommendation**

 Minor Revisions Required

 This is a valuable contribution to regional environmental radiation studies although the number of samples considered are very low. With modest revisions to methodological clarity, data presentation, and the inclusion of recent references and geolocation information, the manuscript will meet high academic standards and be a solid resource for further research.

Is the work clearly and accurately presented and does it cite the current literature?

Partly

If applicable, is the statistical analysis and its interpretation appropriate?

Yes

Are all the source data underlying the results available to ensure full reproducibility?

Yes

Is the study design appropriate and is the work technically sound?

Yes

Are the conclusions drawn adequately supported by the results?

Partly

Are sufficient details of methods and analysis provided to allow replication by others?

Partly

Reviewer Expertise:

Environmental Radioactivity, Natural Radioactivity, and Baseline Surveys

I confirm that I have read this submission and believe that I have an appropriate level of expertise to confirm that it is of an acceptable scientific standard.

## References

[1] : Radon and thoron exhalation rate in the soil of Western Haryana, India. *Environ Monit Assess* .2023;195(4) : 10.1007/s10661-023-11046-7 523 10.1007/s10661-023-11046-7 36988758

[2] : Assessment of surface and mass exhalation rate in soil from Diamond City Surat, Gujrat, India. *Journal of Radioanalytical and Nuclear Chemistry* .2024;333(8) : 10.1007/s10967-024-09507-5 4433-4441 10.1007/s10967-024-09507-5

[3] : An assessment of radionuclides level, radon and thoron exhalation rate in hill and field soil of Mahendergarh district in Haryana, India. *Journal of Radioanalytical and Nuclear Chemistry* .2024;333(6) : 10.1007/s10967-024-09494-7 2649-2659 10.1007/s10967-024-09494-7

[4] : Estimation of radionuclides in the soil samples from the uranium mining zone of Sikar, Rajasthan, India. *Journal of Radioanalytical and Nuclear Chemistry* .2024;333(6) : 10.1007/s10967-024-09386-w 3095-3106 10.1007/s10967-024-09386-w
